# Prenatal choline supplementation improves biomarkers of maternal docosahexaenoic acid (DHA) status among pregnant participants consuming supplemental DHA: a randomized controlled trial

**DOI:** 10.1093/ajcn/nqac147

**Published:** 2022-05-16

**Authors:** Kevin C Klatt, Melissa Q McDougall, Olga V Malysheva, Siraphat Taesuwan, Aura (Alex) P Loinard-González, Julie E H Nevins, Kara Beckman, Ruchika Bhawal, Elizabeth Anderson, Sheng Zhang, Erica Bender, Kristina H Jackson, D Janette King, Roger A Dyer, Srisatish Devapatla, Ramesh Vidavalur, J Thomas Brenna, Marie A Caudill

**Affiliations:** Division of Nutritional Sciences, Cornell University, Ithaca, NY, USA; Children's Nutrition Research Center, Center for Precision Environmental Health, Baylor College of Medicine, Houston, TX, USA; Nutritional Sciences and Toxicology, University of California–Berkeley, Berkeley, CA, USA; Division of Nutritional Sciences, Cornell University, Ithaca, NY, USA; Division of Nutritional Sciences, Cornell University, Ithaca, NY, USA; Division of Nutritional Sciences, Cornell University, Ithaca, NY, USA; Faculty of Agro-Industry, Chiang Mai University, Chiang Mai, Thailand; Division of Nutritional Sciences, Cornell University, Ithaca, NY, USA; Division of Nutritional Sciences, Cornell University, Ithaca, NY, USA; Division of Nutritional Sciences, Cornell University, Ithaca, NY, USA; Proteomics and Metabolomics Facility, Cornell University, Ithaca, NY, USA; Proteomics and Metabolomics Facility, Cornell University, Ithaca, NY, USA; Proteomics and Metabolomics Facility, Cornell University, Ithaca, NY, USA; Division of Nutritional Sciences, Cornell University, Ithaca, NY, USA; OmegaQuant Analytics, LLC, Sioux Falls, SD, USA; The Analytical Core for Metabolomics and Nutrition, BC Children's Hospital Research Institute, University of British Columbia, Vancouver, British Columbia, Canada; The Analytical Core for Metabolomics and Nutrition, BC Children's Hospital Research Institute, University of British Columbia, Vancouver, British Columbia, Canada; Cayuga Medical Center, Ithaca, NY, USA; Cayuga Medical Center, Ithaca, NY, USA; Department of Pediatrics, University of Texas, Austin, TX, USA; Division of Nutritional Sciences, Cornell University, Ithaca, NY, USA

**Keywords:** prenatal choline supplementation, pregnancy, docosahexaenoic acid, PEMT pathway, omega-3 polyunsaturated fatty acids, stable isotope

## Abstract

**Background:**

Dietary methyl donors (e.g., choline) support the activity of the phosphatidylethanolamine *N*-methyltransferase (PEMT) pathway, which generates phosphatidylcholine (PC) molecules enriched in DHA that are exported from the liver and made available to extrahepatic tissues.

**Objectives:**

This study investigated the effect of prenatal choline supplementation on biomarkers of DHA status among pregnant participants consuming supplemental DHA.

**Methods:**

Pregnant participants (*n* = 30) were randomly assigned to receive supplemental choline intakes of 550 mg/d [500 mg/d *d0*-choline + 50 mg/d deuterium-labeled choline (*d9*-choline); intervention] or 25 mg/d (25 mg/d *d9*-choline; control) from gestational week (GW) 12–16 until delivery. All participants received a daily 200-mg DHA supplement and consumed self-selected diets. Fasting blood samples were obtained at baseline, GW 20–24, and GW 28–32; maternal/cord blood was obtained at delivery. Mixed-effects linear models were used to assess the impact of prenatal choline supplementation on maternal and newborn DHA status.

**Results:**

Choline supplementation (550 vs. 25 mg/d) did not achieve a statistically significant intervention × time interaction for RBC PC-DHA (*P* = 0.11); a significant interaction was observed for plasma PC-DHA and RBC total DHA, with choline supplementation yielding higher levels (+32–38% and +8–11%, respectively) at GW 28–32 (*P* < 0.05) and delivery (*P* < 0.005). A main effect of choline supplementation on plasma total DHA was also observed (*P* = 0.018); its interaction with time was not significant (*P* = 0.068). Compared with controls, the intervention group exhibited higher (*P* = 0.007; main effect) plasma enrichment of *d3*-PC (*d3*-PC/total PC). Moreover, the ratio of *d3*-PC to *d9*-PC was higher (+50–67%; *P* < 0.001) in the choline intervention arm (vs. control) at GW 20–24, GW 28–32, and delivery.

**Conclusions:**

Prenatal choline supplementation improves hepatic DHA export and biomarkers of DHA status by bolstering methyl group supply for PEMT activity among pregnant participants consuming supplemental DHA. This trial is registered at www.clinicaltrials.gov as NCT03194659.

## Introduction

DHA (22:6n*–*3) is an omega-3 PUFA that plays a critical role in fetal development (1, 2). The hepatic export of DHA into circulation is linked to the synthesis of phosphatidylcholine (PC) by phosphatidylethanolamine *N*-methyltransferase (PEMT). The PEMT pathway in the liver generates PC molecules enriched in DHA (PC-DHA) that can subsequently be incorporated into VLDLs for export into plasma and delivery to extrahepatic tissues (including the placenta). Transgenic mice lacking PEMT show substantially reduced DHA concentrations in plasma (3) and limited accumulation of DHA in fetal brains of pups born to PEMT-deficient dams (4). Consistent with animal models, previous work from our laboratory has highlighted the importance of PEMT during human pregnancy by demonstrating biomagnification of PEMT-derived PC in fetal cord blood relative to maternal blood at delivery (5).

Dietary methyl donors, such as choline, are essential to support the synthesis of the universal methyl donor, *S*-adenosylmethionine (SAM), utilized by methyltransferases to catalyze the transfer of methyl groups (-CH_3_) to form their respective products. Thus, the activity of the PEMT pathway, and by extension the availability of DHA to extrahepatic tissues, is intrinsically linked to dietary methyl donor and choline intakes. In women of reproductive age with low choline intake (<50 mg/d)–induced organ dysfunction, the proportion of plasma PC containing DHA (DHA as a % of total PC fatty acids) is significantly reduced (6). Conversely, a high-choline intake (930 vs. 480 mg/d), co-administered with 200 mg DHA/d for 12 wk, upregulated PEMT activity in women of reproductive age (5), and led to greater (*P* < 0.001) erythrocyte PC-DHA enrichment and a more rapid increase in plasma PC-DHA (7). An adequate supply of methyl donors for optimal PEMT activity may be particularly important during pregnancy. The promoter region for the *PEMT* gene contains an estrogen response element (8), rendering PEMT activity sensitive to the dramatic increase in circulating estrogen during the latter half of pregnancy. Notably, this increase in PEMT activity coincides with the accumulation of DHA and other long-chain PUFAs in fetal brain (9).

As a major consumer of SAM, it is unsurprising that, during the timing of this large increase in estrogen and PEMT activity, plasma biomarkers of choline-derived methyl donors are reduced in human pregnancy (10), despite “adequate” choline intakes. A higher choline intake (930 vs. 480 mg/d), administered during the third trimester of pregnancy, has been shown to *1*) attenuate this decline (10), *2*) restore choline partitioning between betaine and cytidine diphosphate (CDP)-PC pathways to concentrations observed in nonpregnant women (5), and *3*) enhance the generation of PEMT-derived lysophosphatidylcholine (LPC) that is enriched with DHA (11), and preferentially taken up by fetal brain (12,13). Nonetheless, this higher level of choline intake among third-trimester pregnant women failed to increase PC-DHA (7) in circulating erythrocytes, possibly because PEMT is already operating at maximal capacity in the last third of gestation. Whether prenatal choline supplementation would influence circulating PC-DHA if administered prior to maximal PEMT capacity, or for a longer duration, is unknown. To fill this gap, we investigated the effect of prenatal choline supplementation, administered throughout the second and third trimesters of pregnancy, on biomarkers of DHA status among pregnant participants consuming supplemental DHA.

## Methods

### Ethical approval

Ethical approval was obtained from the Institutional Review Boards for Human Study Participant Use at Cornell University in Ithaca, New York (USA), and at Cayuga Medical Center in Ithaca, New York (the hospital where participants delivered their infants). All participants gave written informed consent before enrollment.

### Participant recruitment and eligibility

Pregnant participants were recruited between October 2017 and April 2019 at maternity clinics throughout the Ithaca, New York, region using flyers. Screening of overall health of the prospective participants was conducted via online questionnaires, which included a short version of the NIH Diet History Questionnaire (DHQ-III; https://www.nal.usda.gov/fnic/dietary-assessment-instruments-research) to estimate usual choline intake, and a validated DSM DHA/EPA food-frequency questionnaire (FFQ; kindly provided by DSM Nutritionals, Inc) to assess omega-3 fatty acid intake. Eligible participants were 12–16 wk pregnant at the beginning of the study, between age 21 and 40 y, had a prepregnancy BMI (in kg/m^2^) <32, were intending to deliver at Cayuga Medical Center, and were willing to comply with the study protocol. Participants were excluded if they had usual dietary DHA intakes exceeding 400 mg/d (based on FFQ), had usual dietary choline intakes exceeding 450 mg/d (based on DHQ-III), or reported having cardiovascular disease, cancer, type 1 or 2 diabetes, gastrointestinal disorders, gallbladder disease, kidney disease, liver disease, or anemia (based on a health questionnaire). Additional exclusion criteria included the following: use of prescription medications known to affect liver function; presence of more than 1 fetus; unwillingness to donate placenta; self-reported tobacco, recreational drug, or alcohol use during gestation; or presence (or development) of pregnancy-associated complications (e.g., preeclampsia, gestational diabetes). The latter exclusion criterion impacted participants already enrolled in the trial and randomly assigned to the treatment; participants were dismissed from the trial out of an abundance of caution, as the impact of choline supplementation on the risk of exacerbating pregnancy-associated complications is unknown.

### Study design

The present study was a single-center, randomized, double-blind, parallel-group choline intervention study designed to investigate the effects of prenatal choline supplementation on DHA status in healthy free-living pregnant participants consuming supplemental DHA (NCT03194659). The intervention of 550 mg supplemental choline/d or control (25 mg supplemental choline/d) was initiated at gestational age 12–16 weeks and continued until delivery; throughout this period, all participants received 200 mg supplemental DHA/d. Data obtained during the study were recorded in a dedicated online secure database developed by Cornell Institute of Social and Economic Research (CISER).

### Randomization and masking

Eligible participants (*n* = 33) were enrolled into the study by MQM on a rolling basis in a parallel-arm design. A simple 1:1 randomization scheme was generated by KCK using a web-based random-number generator assigning participants to either group A or group B; study staff (OVM) not interacting with participants replaced group A or group B with the intervention or control. Both groups consumed a grape juice cocktail that provided either 550 mg supplemental choline/d (intervention) or 25 mg supplemental choline/d (control). Supplemental doses in this study aimed to mirror previous studies demonstrating improvements in choline and methyl donor status markers (5, 10) as well as cognitive benefits from supplementation (14). Preparation of the grape juice cocktail was handled by study staff not interacting with study participants (OVM) to maintain personnel blinding. The grape juice cocktail was served in 15-mL color-coded conical tubes to conceal the intervention assignment from the participant.

### Procedures

#### Choline supplement

Participants consumed a grape juice cocktail containing 550 mg choline/d (intervention) or 25 mg choline/d (control) throughout the duration of the study. The 550 mg choline dose for the intervention group was composed of 500 mg unlabeled choline (*d0*-choline) and 50 mg methyl-*d9*-choline (*d9*-choline). The 25-mg choline dose for the control group was in the form of *d9*-choline only. The low-dose deuterium-labeled choline tracer (50 mg *d9*-choline for the intervention group and 25 mg *d9*-choline for the control group) was administered to provide insights into the use of choline-derived methyl groups for the synthesis of PC by the PEMT pathway (described in the Results section). Doses were adjusted for the intervention and control to attempt to account for dilution of the label in the endogenous choline pool that was simultaneously increased with the intervention.

The choline supplements were prepared in the Human Metabolic Research Unit (HMRU) at Cornell University. First, stock solutions of *d0*-choline and *d9*-choline were prepared separately by dissolving choline chloride (Balchem, Inc.) or *d9*-choline chloride (Cambridge Isotope Laboratories, Inc.) in Milli-Q (MilliporeSigma) water to produce 250 mg/mL *d0*-choline and 50 mg/mL *d9*-choline solutions. The stock solutions were filtered and stored at 4°C for up to 1 y; tests conducted during this period showed excellent stability with no detectable loss through time. Next, the choline and control supplements were assembled by aliquoting 2 mL of the *d0*- choline stock solution (intervention group), 1 mL of the *d9*-choline stock solution (intervention group), or 0.5 mL of the *d9*-choline stock solution (control group) into 15-mL sterilized polystyrene tubes. The supplement tubes were filled with grape juice cocktail (Welch's) and labeled in a manner that was indistinguishable by both the participants and personnel who interacted with the participants. Finally, all supplement tubes were packaged in Ziplock (SC Johnson) bags labeled with the participant ID numbers and kept frozen in a food-only −20°C freezer prior to pick-up. Testing of the supplements that were kept for 2 mo at room temperature, or inside a home refrigerator, showed excellent stability with no detectable loss over time.

#### Study protocol

Participants were required to visit the HMRU on 3 separate occasions. The first visit was during gestational week (GW) 12–16, while the second (GW 20–24) and third (GW 28–32) visits were every 8 wk thereafter. At each study visit, participants received their daily supplements, which included the grape juice choline cocktail tubes, a 200-mg DHA supplement [Nature's Way EfaGold Neuromins 200 mg DHA (plant source); DSM Nutritional Products], and an over-the-counter prenatal vitamin/mineral supplement (Nature Made Prenatal Tablet; Pharmavite LLC). Participants were instructed to consume the supplements and grape juice cocktail daily, and at the latter 2 HMRU visits, to return supplement containers as a means to assess adherence to the study protocol. Participants were also asked to discontinue any supplements outside of those provided by this study. Self-selected diets were consumed by participants throughout the study period.

At each study visit, participants provided an 8–10-h fasting blood sample, and height and weight measurements were recorded. At visits 2 and 3, participants completed a follow-up health questionnaire to assess changes in health status and medication/supplement use. At visit 1 (baseline), participants completed the National Cancer Institute's Automated Self-Administered 24-hour (ASA24) Dietary Recall, under the supervision of research personnel. Additionally, participants completed 2 to 4 self-administered 24-h dietary recalls (ASA24) throughout the course of their second and third trimesters, prior to delivery, to assess usual mean nutrient intakes.

At delivery, nonfasting maternal blood, placenta, and fetal cord blood were collected. In addition, maternal and newborn information was obtained from medical charts. Maternal information included due date, complications during pregnancy, and complications during labor or delivery. Newborn information included the date and mode of delivery, gestational age, birth weight, sex, and Apgar score.

#### Sample collection and processing

Fasting blood samples (8–10 h) were collected at each of the 3 HMRU visits in one 10-mL serum separator gel and clot-activator tube (SST Vacutainer; Becton, Dickinson, and Company), three 10-mL EDTA-coated tubes (Becton, Dickinson, and Company), and one 5-mL EDTA-coated tube (Becton, Dickinson, and Company). The three 10-mL EDTA-coated tubes containing whole blood were placed on ice immediately following collection and were centrifuged within 90 min at 2000 × *g* for 15 min at 4°C. Plasma was removed for metabolite measurements and buffy coat was removed and mixed with 50 μL DMSO for DNA extraction. Remaining RBCs were either stored or washed 3 times with PBS. The 5-mL EDTA-coated tube, which provided whole blood for the complete blood counts, was processed within 60 min by research personnel in the Cornell Human Nutritional Chemistry Laboratory. The serum separator tube (SST) blood, which provided serum for the blood chemistry profile, was kept at room temperature, allowed to clot, and centrifuged at 1500 × *g* for 15 min at room temperature. All of the biological samples were dispensed into 1.8-mL cryostat vials (CryoTube; NUNC) and stored at −80°C.

Maternal blood and cord blood samples were collected at delivery. Maternal blood samples were collected into two 10-mL EDTA-coated tubes and one 10-mL SST tube at the hospital within 1 h of delivery. Cord blood samples were collected into two 4-mL EDTA-coated tubes at the time of delivery. After collection, maternal and cord blood samples were refrigerated at 4°C, and processed as described previously within 4 h.

The placenta was also obtained at delivery, weighed, and processed at the hospital within 90 min of delivery. After removal of the amnion, 16 full-thickness tissue biopsies (0.5 × 0.5 × 0.5 cm) were taken from 4 separate locations (i.e., the placenta was visually divided into 4 quadrants). Samples were rinsed with PBS immediately. The other samples were flash-frozen in liquid nitrogen, placed in a cryostat tube, and stored temporarily in a canister containing liquid nitrogen. In the laboratory, all samples were stored at −80°C. Prior to using the placental samples for analytical measurements (i.e., total DHA), a homogenous representation was prepared by taking 1 piece of placenta from each quadrant and powderizing in liquid nitrogen using a Bessman Tissue Pulverizer (Fisher Scientific).

### Analytical measurements

#### PC-DHA measurements in RBCs

In our a priori planned analysis of RBC PC-DHA (% fatty acids in RBC PC), we proposed utilizing stable-isotope dilution LC–tandem MS (LC-MS/MS); however, simultaneous measurement of individual PC species containing DHA and total PC (to facilitate expressing % RBC PC composition) in the RBC sample matrix proved technically challenging and exhibited low reproducibility. As such, samples were sent to the Analytical Core for Metabolomics and Nutrition (BC Children's Hospital Research Institute), which utilizes a well-validated method whereby PC is separated from the other lipids by HPLC followed by analysis of the PC fatty acids using gas–LC (GLC) (15). Briefly, RBC lipids were extracted from washed RBCs using a modification of the method from Rose and Oklander (16), and PC was separated using a Waters 2690 HPLC, a quaternary solvent system of hexane, methanol, acetone, and isopropanol, and a Waters YMC-Pack DIOL column. The HPLC column eluant was split 10:90, after which lipids were detected and quantified by a Waters 2424 evaporative light scattering detector and recovered with use of a Waters FCIII fraction collector. Following the evaporation of solvents under nitrogen, fatty acids were converted to methyl esters, separated, and quantified by GLC. RBC PC-DHA is expressed as a percentage of the total fatty acids in PC. The interassay CV for RBC PC-DHA (as a percentage of the total fatty acids in PC) was 6.4% based on in-house RBC controls.

#### PC-DHA measurements in plasma

PC-DHA concentration of plasma (micromoles/liter) was analyzed using stable-isotope dilution LC-MS/MS at Cornell University's Biotech Proteomics and Metabolomics Facility. Briefly, plasma (20 μL) was transferred to 1.5 ml Eppendorf vials (VWR20170-038), to which 2.4 nmol *d7*-PC 15:0–18:1 (Avanti Polar Lipids) in 0.2 mL methanol:chloroform (2:1 vol:vol) was added as an internal standard. The samples were mixed and incubated overnight at −20°C. On the following day, samples were centrifuged at 1500 × *g* at 4°C for 5 min, and the supernatant was collected and transferred into another Eppendorf tube without disturbing the solid phase. To the pellet, 0.25 mL of methanol:chloroform:water (2:1:0.8) was added, followed by mixing on a vortex, and centrifugation. The resulting supernatant was collected and combined with the first supernatant. To the pooled supernatant, chloroform (100 μL) and optima water (100 μL) were added; samples were mixed and then centrifuged. Using a long gel-loading pipette tip, the lower chloroform phase was transferred into glass vials, dried in a speed vac, and reconstituted with 200 μL methanol:chloroform (6:1 vol:vol) for subsequent LC-MS/MS analysis.

The LC-MS/MS analysis of PC-DHA species was performed using a Syncronis Silica column (150-mm length × 2.1-mm internal diameter, 5-µm particle size; ThermoScientific) in an Exion LC system coupled with a Sciex X500B QTOF mass spectrometer. PC-DHA species were separated under step gradient conditions with a flow rate of 400 μL/min. The column temperature was kept at 30°C. The mobile phase (MP) consisted of MPA (400 mL acetonitrile:127 mL water:68 mL ethanol:3 mL 1 M ammonium acetate in water:2 mL concentrated glacial acetic acid) and MPB (250 mL acetonitrile: 250 mL water: 42 mL ethanol: 13.5 mL 1 M ammonium acetate in water: 9 mL of concentrated glacial acetic acid). The following step gradient was used for the separation and analysis of PC-DHA species: 5% MPB from 0 to 3 min; 30% MPB from 3 to 10 min; 60% MPB from 10 to 14 min; 100% MPB from 16 to 17 min; 5% MPB from 17 to 19 min. Total run time was 19 min and retention times at 1.90 min, 1.98 min, 2.04 min, and 2.19 min were observed for PC 18:0-DHA, PC 18:1-DHA, PC 16:0-DHA, and *d7*-PC 15:0–18:1 (internal standard [IS]). The injection volume was 10 μL for each of the standards and samples. The autosampler temperature was kept constant at 5°C.

The Sciex X500B QTOF mass spectrometer with an ESI Turbo V™ source was operated in the negative ion mode for this analysis. The electrospray voltage was set at 4.5 kV and the temperature of the heated capillary was set at 350°C. It was operated under the Ion Source gas 1 and 2 at 30 psi, curtain gas at 30 (arbitrary units), and collision gas (CAD) gas at 7 (arbitrary units). The declustering potential was set to −35 V with an accumulation time of 0.25 s. The MS full-scan measurement was done from *m/z* 100 to *m/z* 1000 in profile mode followed by multiple reaction monitoring (MRM) high resolution (HR) scan acquired from 0 min to 21 min at collision energies of −40 V for each analyte. The acetate adduct negatively charged ion of each analyte was selected as the precursor ion. For the internal standard (*d7*-PC 15:0–18:1), loss of 18:1 fatty acid from acetate adduct was used as a product ion for an MRM transition pair at *m/z* 811.62/288.2924. Negative ion MRM transition pairs were monitored at *m/z* 864.57/327.2340 for PC 16:0-DHA, *m/z* 892.61/327.2340 for PC 18:0-DHA, and *m/z* 890.59/327.2340 for PC 18:1-DHA. Loss of DHA fragments (*m/z* 327.2340) of acetate adducts from each PC-DHA species was the major fragment used in the MRM HR method for quantitation. The data were acquired using Sciex OS 2.0 software and the quantitation ratio PC-DHA:IS was calculated for each sample by integrating the peak areas by MQ4 Integration *Algorithm* of each analyte using MRM transitions in the same software.

Calibration curves standard solutions were prepared by serial dilutions of the standards (PC 16:0-DHA, PC 18:0-DHA; Avanti Polar Lipids), mixed with the internal standard (*d7*-PC 15:0–18:1) at a concentration of 12 nmol/mL in 6:1 MeOH:CHCl_3._ The calibration curves ranged from 50 to 5000 pmol for PC 16:0-DHA and PC 18:0-DHA (*R*^2^ >0.99). The lower limit of detection and lower limit of quantitation, determined based on the signal to noise ratio (S/N) in PC DHA standards solutions, were 0.15 pmol and 0.5 pmol, respectively. All area ratios of PC 16:0-DHA and PC 18:0-DHA quantified and calculated (peak area of analyte/peak area of IS) in the plasma samples fell within the linear dynamic range of calibration standards. Since no commercial standard was available for PC 18:1-DHA, the standard curve for PC 16:0-DHA was used to derive absolute concentrations of PC 18:1-DHA (which was 1.3% of the total PC-DHA concentration).

To validate the LC-MS/MS method for quantitation of PC-DHA species in plasma samples, 4 quality assurance (QA) samples were analyzed several times to determine the intra-day and inter-day variation for assay precision. For plasma samples, which were extracted and analyzed at the same time, intra-assay CVs were 3.1% (PC 16:0-DHA) and 2.1% (PC 18:0-DHA). Recovery yield for PC-DHA extraction method was also determined by spiking the 2.4 nmol IS in 20 μL of plasma samples before and after extraction of PC-DHA species, and it was found to be 92%. Matrix effect was also calculated as 22% for the internal standard. This value represents a loss of 22% of IS peak area (ion suppression) in plasma samples compared with pure solvent due to alteration in ionization efficiency. PC-DHA in plasma is expressed as the sum of PC 16:0-DHA + PC 18:0-DHA + PC 18:1-DHA. Plasma PC-DHA isotopologues could not be reliably quantified due to the presence of co-eluting metabolites of the same monitored *m/z* as the predicted *m/z* (+3, +9); the pattern of labeling would be expected to minimally influence overall estimates of plasma PC-DHA (<3%) and similarly affect the intervention and control arms, limiting the bias introduced in our effects of choline supplementation.

#### Total DHA measurements in RBCs and plasma

DHA composition of washed RBCs (% total fatty acids) and concentrations in plasma (micrograms/milliliter) were analyzed by GC coupled to a flame ionization detector at OmegaQuant®, as previously described (17, 18). Briefly, RBCs and plasma were transferred to screw-cap glass vials, after which 14% boron trifluoride (Sigma-Aldrich) and hexane (EMD Chemicals) were added to RBCs, and BTM (methanol containing 14% boron trifluoride, toluene, methanol; 35:30:35 vol:vol:vol; Sigma-Aldrich) was added to plasma. Placenta samples were weighed into screw-cap glass vials that contained tritricosanoin as an internal standard (tri-C23:0 triglyceride) (NuCheck Prep), homogenized, and subsequently subjected to a modified Folch extraction. A portion of the organic layer was transferred to a new screw-cap glass vial and dried in a speed vac. BTM was then added to the dried placenta samples. Next, sample vials (RBCs, plasma, and placenta) were briefly mixed on a vortex and heated in a hot bath at 100åC for 45 min. After cooling, HPLC grade water was added, and vials were recapped, mixed on a vortex, and centrifuged to separate layers. Aliquots of the hexane layer were subsequently transferred to GC vials.

GC was carried out using a GC-2010 Gas Chromatograph (Shimadzu Corporation) equipped with an SP-2560, 100-m fused silica capillary column (0.25-mm internal diameter, 0.2-μm film thickness; Supelco). Fatty acids were identified by comparison with a standard mixture of fatty acids (GLC OQ-A; NuCheck Prep), which was also used to determine individual fatty acid calibration curves. The following 24 fatty acids (by class) were identified: saturated (14:0, 16:0, 18:0, 20:0, 22:0, 24:0), *cis* monounsaturated (16:1, 18:1, 20:1, 24:1), *trans* unsaturated (16:1, 18:1, 18:2), *cis* n–6 polyunsaturated (18:2, 18:3, 20:2, 20:3, 20:4, 22:4, 22:5), and *cis* n–3 polyunsaturated (18:3, 20:5, 22:5, 22:6). Fatty acid composition, specifically DHA for this study (*cis* C22:6, n–3), was expressed as a percentage of total identified fatty acids. The interassay CV for total DHA in RBCs, plasma, and placenta was <5% based on in-house controls.

#### Maternal genotypes

Single nucleotide polymorphisms in the *PEMT* gene (*PEMT* rs7946, *PEMT* rs4646343), which may influence PEMT activity (19), were determined as previously described (20, 21) so that these variables could be considered in our statistical models if differentially distributed between the intervention and control groups.

#### Choline and PC measurements

Stable-isotope dilution LC-MS/MS was used to measure plasma free choline (22, 23), plasma PC (23, 24), and plasma enrichment percentages of *d3*-PC and *d9*-PC (23, 24). Intra- and interassay CVs were ≤3% for choline and ≤6% for PC.

#### Hematology and chemistry panels

The complete blood count and blood chemistry profiles were performed at the Human Nutritional Chemistry Service Laboratory at Cornell University.  Hematology analysis was conducted using a Beckman-Coulter AcT Diff2 coulter counter; serum albumin, alanine aminotransferase (ALT), aspartate aminotransferase (AST), glucose, total cholesterol, HDL cholesterol, LDL cholesterol, and triglycerides were measured using an automated chemistry analyzer (Dimension Xpand Plus; Siemens Healthcare Diagnostics).

### Study outcomes

The primary outcome of this study was DHA-containing PC species in circulating maternal RBCs. PC species containing DHA (PC-DHA) at either the sn-1 or sn-2 positions are found circulating in RBCs and have been shown to be responsive to dietary choline intake among women of reproductive age (7). Secondary outcomes included total DHA in maternal RBCs, an established surrogate for tissue DHA concentrations (25), as well as maternal plasma measures of total DHA, PC-DHA, and PC (unlabeled and isotopically labeled forms). All outcomes were measured at gestational age 12–16 wk (baseline, study visit 1), gestational age 20–24 wk (study visit 2), gestational age 28–32 wk (study visit 3), and at delivery (gestational age 36–42 wk). In addition, we included measurements of total DHA in fetal RBCs and plasma, as well as the placenta. Exploratory/descriptive outcomes included the following: *1*) self-reported dietary intakes of choline and DHA assessed at baseline (visit 1) and throughout the second and third trimesters of pregnancy; *2*) maternal clinical parameters [i.e., white blood cell (WBC) counts, RBC counts, and hemoglobin assessed at visit 1, visit 2, and visit 3; serum concentrations of albumin, ALT, AST, glucose, total cholesterol, HDL cholesterol, LDL cholesterol, and triglycerides assessed at visit 1, visit 3, and delivery]; and *3*) newborn characteristics (i.e., infant sex, weight, length, head circumference, Apgar scores, and placental weights).

### Statistical analysis

The study sample size was based on previous results among nonpregnant women (7), whereby means and SDs of erythrocyte PC-DHA for 2 levels of choline intake yielded a Cohen's *d* effect size of 1.1, after accounting for statistically significant covariates. G*Power 3.1 (Henrich Heine Universitat Dusseldorf) was used to model estimated sample sizes; accordingly, a sample size of 15 for each group (*n* = 30 total) was required to have a power of 80% at an ɑ of 0.05 utilizing a *t* test between the control and intervention groups. Participants were continuously enrolled until 30 participants had completed the trial; all participants who completed the trial (i.e., delivery data were available) were included in all analyses by intention to treat (ITT; a modified ITT excluding participants who were excluded due to the development of pregnancy-related pathologies).

Differences between intervention and control groups for participant characteristics and baseline measures, as well as infant characteristics, were assessed using Wilcoxon rank-sum tests (continuous variables) or chi-square test of independence (categorical variables). Q-Q plots and histograms of DHA-related outcomes were visually assessed for normality; the Shapiro–Wilk test and skewness was used to assess normality and symmetry of the data. Mixed linear models that controlled for baseline measures (with the exception of labeled PC) were used to assess the effect of the choline intervention on maternal outcomes (i.e., RBC PC-DHA, plasma PC-DHA, RBC total DHA, plasma total DHA, plasma free choline, plasma PC, plasma lipids, and clinical parameters) throughout the course of the study. In addition to including baseline values of the respective metabolite, these models included intervention arm, time, and their interaction term (intervention × time) as fixed effects, and participant ID as a random effect (random intercepts). These model estimates are depicted in the graphs and tables and reported in the primary text. Additional sensitivity analyses were conducted to assess the robustness of the total DHA outcomes, due to their widespread use as a markers of DHA status. These sensitivity analyses included models without adjustment for baseline (unadjusted model), as well as a model that considered additional covariates hypothesized to influence the intervention effects and/or DHA outcomes, specifically age, prepregnancy BMI, and calculated duration of intervention exposure (i.e., gestational age at delivery – gestational age at study entry) (fully adjusted models). To assess the impact of choline intake on fetal DHA outcomes, linear models that included intervention arm and baseline maternal RBC DHA as fixed effects were used. For self-reported intakes of dietary choline and dietary DHA, *P* values were derived from Hodges-Lehman-Sen estimations for nonparametric comparison of medians.

All statistical analyses and graphs were generated using the R statistical program language and environment, version 3.6.1 (R Foundation for Statistical Computing). Graphs were developed using “ggplot2,” plotting estimated marginal means and CIs derived from (mixed) linear models. Statistical significance was set at *P* ≤ 0.05. For outcomes with a significant interaction term, Tukey's honestly significant difference post hoc test was used to assess the significance of differences between the subgroups.

## Results

### Participant characteristics

**FIGURE 1:**
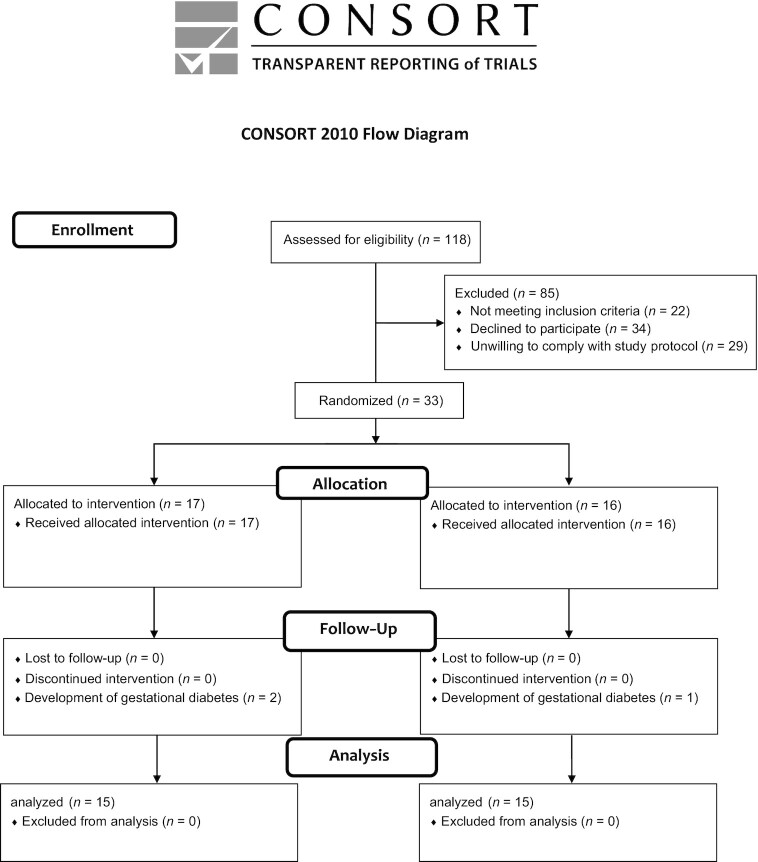
Flowchart of the study. CONSORT, Consolidated Standards of Reporting Trials.

A total of 118 potential participants were prescreened by completing online questionnaires. Of these potential participants, 85 were excluded for the following reasons: did not meet eligibility criteria (*n* = 22), declined to participate (*n* = 34), or were unwilling to comply with the study protocol (*n* = 29). The remaining 33 eligible subjects were randomly assigned to the choline intervention group (*n* = 17) or control group (*n* = 16). Of these 33 participants, 3 developed gestational diabetes during the intervention trial and were discontinued. Thus, 30 participants (*n* = 15 in the intervention group and *n* = 15 in the control group) were included in the ITT analyses. **Figure 1** depicts the flowchart of the study. **Table 1** presents baseline demographics and baseline measures of the participants included in the ITT analysis by group allocation. Of note, participants’ reported choline intakes were similar to representative samples of choline intake within the North American population (26).

**TABLE 1:** Participant characteristics and baseline measures^1^

	Intervention (*n =* 15)	Control (*n =* 15)
Age, y	33 (3)	31 (4)
Prepregnancy BMI, kg/m^2^	22.3 (2.1)	25.1 (4.1)
Self-reported maternal race, count		
White	13	15
Non-White	2	0
Baseline gestational age, wk	15 (1)	15 (1)
Enrollment duration, d	171 (17)	169 (11)
Baseline RBC PC-DHA, % of RBC PC total fatty acids	2.99 (0.90)	2.69 (0.42)
Baseline plasma PC-DHA, μmol/L	100 (40)	86 (22)
Baseline RBC-DHA, % of total fatty acids	6.09 (1.09)	5.89 (0.07)
Baseline plasma DHA, μg/mL	94 (31)	83 (13)
Self-reported baseline dietary DHA intake, median (Q1–Q3), mg/d	14 (1–85)	36 (10–70)
Self-reported baseline dietary choline intake, median (Q1–Q3), mg/d	320 (204–384)	364 (326–506)
Baseline DHA supplement use, count		
No	9	9
Yes	6	6
*PEMT* rs7946 genotype, count		
Noncarrier	8	5
Carrier	7	10
*PEMT* rs4646343 genotype, count		
Noncarrier	4	5
Carrier	11	10

1 Values are mean (SD) unless noted otherwise. PC, phosphatidylcholine; *PEMT*, phosphatidylethanolamine N-methyltransferase; Q, quartile.

Participant adherence to the study protocol was >99%, based on the number of returned juice containers (containing the choline supplement) and the number of returned DHA capsules. Further, mean concentrations of plasma free choline, which have been shown to be responsive to choline supplementation during pregnancy (10), were significantly higher in this cohort in the choline intervention (vs. control) group, with between-group differences detected at visit 2, visit 3, and delivery (27).

The intervention trial was well tolerated, and participant-reported in-trial events were similarly distributed between the 2 groups (**Supplemental Table 1**). The choline intervention was found to associate in a statistically significant manner with some of the clinical parameters assessed with the complete blood count and blood chemistry profile (i.e., WBC counts, RBC counts, and hemoglobin; **Supplemental Table 2**); however, mean concentrations of the clinical parameters remained within the normal range for both the intervention and control groups (28, 29,28, 29). No differences (*P* ≥ 0.13) between the groups were observed for serum concentrations of albumin, ALT, AST, and glucose at any of the study time points (GW 20–24 through delivery). For newborn characteristics, no statistically significant effects of the choline intervention were observed on neonatal weight (*P* = 0.4), length (*P* = 0.8), head circumference (*P* = 0.7), Apgar scores (*P* = 0.1), or placental weights (*P* = 0.4) (**Supplemental Table 3**). Last, median intakes of self-reported dietary DHA (17 and 34 mg/d for intervention and control, respectively; *P* = 0.41) and self-reported dietary choline (353 and 377 mg/d for intervention and control; *P* = 0.15) did not differ between the groups throughout the duration of the study (**Supplemental Table 4**).

### Study outcomes

#### PC-DHA

##### PC-DHA in maternal RBCs

**FIGURE 2:**
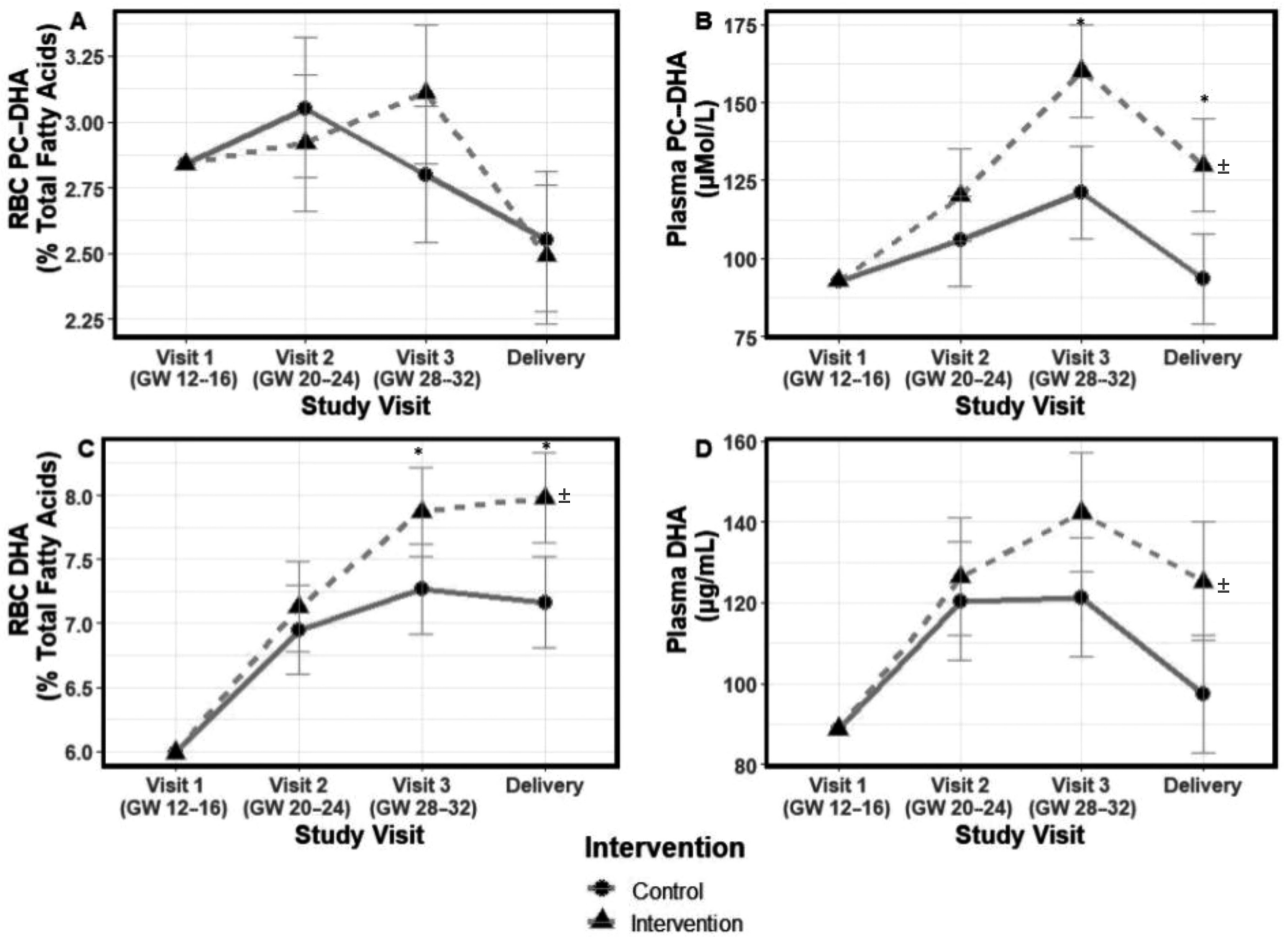
Effect of prenatal choline supplementation (*n* = 15 per group) on maternal RBC PC-DHA (A), maternal plasma PC-DHA (B), maternal RBC total DHA (C), and maternal plasma total DHA (D) among pregnant participants consuming 200 mg supplemental DHA/d. The effect of prenatal choline supplementation on maternal RBC and plasma DHA outcomes was assessed using mixed linear models that included choline intervention × time interaction terms as well as baseline values for the respective outcome. Estimated marginal means and 95% CIs derived from the baseline adjusted models are shown. Statistically significant differences (*P* < 0.05) between the choline intervention and control values at a study time point are indicated by “*” for models with a significant (*P* < 0.05) choline intervention × time interaction term (i.e., maternal plasma PC-DHA and maternal total RBC DHA) and by “^±^” at study end for models with a significant main effect of the choline intervention (i.e., maternal plasma total RBC DHA). GW, gestational week; PC, phosphatidylcholine.

Significant effects of time (*P* < 0.000084) were observed for maternal RBC PC-DHA; however, no significant effects of the choline intervention (*P* = 0.77) or its interaction with time (*P* = 0.11; choline intervention × time) were detected (**Figure 2**A). Nonetheless, RBC PC-DHA values were non–normally distributed and recalcitrant to various transformations (log; square root; arcsine). Two participants at delivery (1 control, 1 intervention) were significant outliers (>2 SDs) and exhibited atypical responses: the control participant exhibited a 70% increase and the intervention participant exhibited a 78% decrease in RBC PC-DHA from GW 28–32 to delivery, several times greater than the average 15% decrease between these 2 time points for the cohort. Sensitivity analyses removing these 2 individuals’ data points yielded a significant choline intervention by time interaction (*P* = 0.010); RBC PC-DHA (as a percentage of RBC PC total fatty acids) was higher in magnitude in the intervention at GW 28–32 [3.2% (95% CI: 2.9, 3.5%) vs. 2.7% (95% CI: 2.4, 3.0%); *P* = 0.05] and delivery [2.7% (95% CI: 2.4, 3.0%) vs. 2.3% (95% CI: 2.0, 2.6%); *P* = 0.16] relative to the control group.

As our intervention was powered from a trial without RBC PC-DHA data from delivery, a more variable time point due to its nonfasting nature and the metabolic milieu of parturition, we considered models omitting this time point as well. Significant choline intervention × time interactions were observed in such models (*P* < 0.00005). RBC PC-DHA tended to be higher in the intervention at GW 28–32 [3.1% (95% CI: 2.9, 3.3%) vs. 2.8% (95% CI: 2.6, 3.0%); *P* = 0.07] relative to the control group.

##### PC-DHA in maternal plasma

Significant effects of the choline intervention (*P* = 0.0030), time (*P* < 0.00001), and their interaction (*P* = 0.012; choline intervention × time) were detected for maternal plasma PC-DHA (Figure 2B). Plasma PC-DHA exhibited an inverted U-shaped response across the study, increasing through GW 28–32 in both the intervention and control groups before declining at delivery; however, a significantly higher plasma PC-DHA concentration (micromoles/liter) was achieved in the intervention group at GW 28–32 [160 (95% CI: 145, 175) vs. 121 (95% CI: 106, 136) μmol/L; *P* = 0.0005] and delivery [130 (95% CI: 115, 145) vs. 94 (95% CI: 79, 108) μmol/L; *P* = 0.0012].

#### Total DHA

While RBC PC-DHA was the primary outcome of this investigation (due to previous trial evidence that choline supplementation influenced plasma and RBC PC-DHA), choline's influence on PC-DHA is ultimately physiologically meaningful to the extent that it contributes to total DHA availability in the plasma and total DHA concentrations in cell membranes (found across all phospholipid species). Indeed, PC-DHA in plasma and in the cell can be remodeled and facilitate DHA entering additional lipid pools (e.g., LPC, cholesterol esters, phosphatidylethanolamine). Total DHA in RBCs (% of total RBC fatty acids) is a well-validated indicator of tissue DHA concentrations (i.e., DHA status), consistently linked to health outcomes (including pregnancy-related health outcomes), and thus, the most physiologically relevant outcome of interest in relation to choline supplementation's impact.

##### Total DHA in maternal RBCs

Significant effects of the choline intervention (*P* = 0.009), time (*P* < 0.001), and their interaction (*P* = 0.002; choline intervention × time) were detected for maternal RBC total DHA (Figure 2C). Specifically, RBC total DHA (as a percentage of total fatty acids) increased throughout the study in both the intervention and control groups; however, a significantly higher level was achieved in the intervention group at GW 28–32 [7.9% (95% CI: 7.5, 8.2%) vs. 7.3% (95% CI: 6.9, 7.6%); *P* = 0.008] and at delivery [8.0% (95% CI: 7.6, 8.3%) vs. 7.2% (95% CI: 6.8, 7.5%); *P* = 0.0005]. The statistically significant effect of the choline intervention on RBC DHA was robustly observed in both unadjusted models and models adjusting for additional covariates (**Supplemental Table 5**).

##### Total DHA in maternal plasma

Significant effects of the choline intervention (*P* = 0.018) and time (*P* = 0.0002) were detected for maternal plasma total DHA; however, the choline intervention × time interaction term did not achieve our threshold for statistical significance (*P* = 0.068) (Figure 2D). While both groups exhibited an inverted U-shaped response through time with an initial increase in plasma total DHA until GW 28–32 followed by a decrease at delivery, a significantly higher plasma total DHA was observed in the intervention (vs. control) group throughout the study period (Figure 2D). The statistically significant effect of the choline intervention on plasma DHA was robustly observed in both unadjusted models and models adjusting for additional covariates (Supplemental Table 5).

##### Total DHA in placenta, cord RBCs, and cord plasma

No significant effects of the choline intervention were detected on placenta DHA [as either a percentage of the total fatty acids (*P* = 0.77) or absolute concentration (micrograms/milligram) (*P* = 0.42)], cord RBC DHA (*P* = 0.12), or plasma DHA (*P* = 0.91) in the model that controlled for baseline concentrations of maternal RBC DHA or in the unadjusted model (**Table 2**).

**TABLE 2:** Effect of prenatal choline supplementation on placental total DHA and newborn total DHA among pregnant participants consuming 200 mg supplemental DHA/d^1^

	Baseline adjusted, mean (95% CI)		
	Intervention (*n =* 13)	Control (*n =* 15)	*P*
Placenta			
DHA, % of total FAs	0.05 (0.05, 0.06)	0.05 (0.05, 0.06)	0.77
DHA, μg/mg	0.43 (0.39, 0.48)	0.46 (0.41, 0.50)	0.42
Cord			
RBC-DHA, % of total FAs	8.3 (7.8, 8.8)	7.9 (7.5, 8.3)	0.12
Plasma DHA, μg/mL	54 (41, 67)	54 (42, 65)	0.91

1 Linear models included the choline intervention term as well as baseline values for maternal RBC DHA. FA, fatty acid.

#### Unlabeled and isotopically labeled PCs in plasma

The use of choline as a methyl donor by the PEMT pathway was assessed by measuring *d3*-PC enrichment (*d3*-PC/total PC) and the ratio of *d3*-PC to *d9*-PC. *d3*-PC is generated by the PEMT pathway when one of the *d3*-labeled methyl groups is used in the SAM-dependent methylation of phosphatidylethanolamine to PC, whereas *d9*-PC is generated by the CDP-choline pathway when the intact choline molecule (*d9*-choline) is used to generate PC (5, 23). Collectively, our results (described herein) are consistent with greater use of choline as a methyl donor by the PEMT pathway and greater PEMT activity in response to the choline intervention.

##### Total PC

**FIGURE 3:**
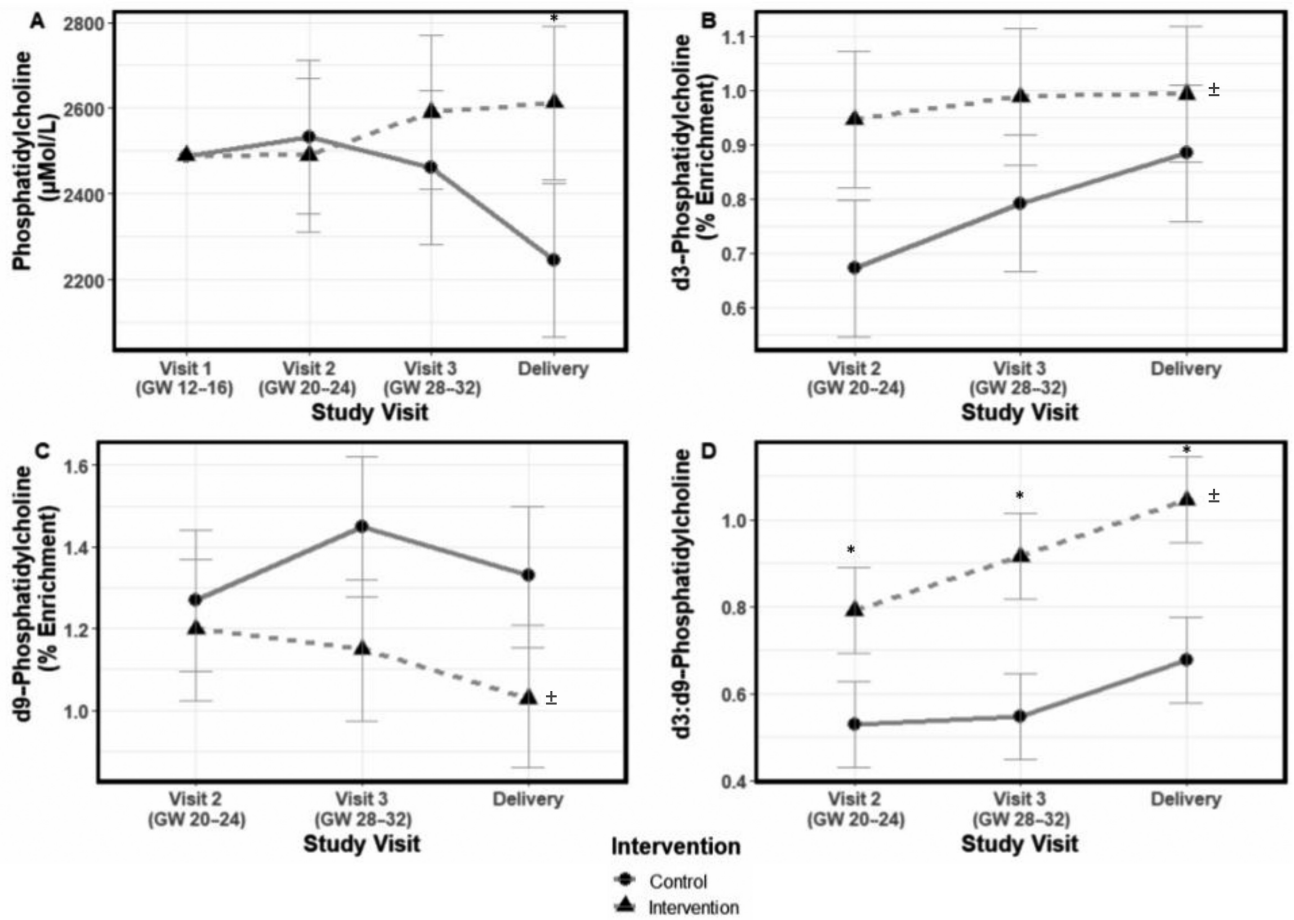
Effect of prenatal choline supplementation (*n* = 15 per group) on maternal plasma total PC (A), *d3*-PC (B), *d9*-PC (C), and *d3*:*d9*-PC (D). The effect of prenatal choline supplementation on maternal total and labeled PC was assessed using mixed linear models that included choline intervention × time interaction terms. Estimated marginal means and 95% CIs are shown; total PC concentrations represented models adjusted for visit 1 plasma PC. Statistically significant differences (*P* < 0.05) between the choline intervention and control values at a study time point are indicated by “*” for models with a significant (*P* < 0.05) choline intervention × time interaction term (i.e., maternal plasma total PC and *d3*:*d9*-PC) and by “^±^” at study end for models with a significant main effect of the choline intervention (i.e., *d3*-PC and *d9*-PC). *d3*, tri-deuterated; *d9*, nona-deuterated; GW, gestational week; PC, phosphatidylcholine.

A significant choline intervention by time interaction term (*P* = 0.05) was detected for maternal plasma total PC (**Figure 3**A) with higher total plasma PC (micromoles/liter) at delivery (*P* = 0.006) in the intervention (vs. control) group [2613 (95% CI: 2434, 2792) vs. 2247 (95% CI: 2068, 2426) μmol/L].

##### *d3*-PC

Significant main effects of the intervention (*P* = 0.007) and time (*P* = 0.045) were detected for maternal plasma *d3*-PC (Figure 3B) with higher plasma *d3-*PC enrichment observed in the intervention (vs. control) group throughout the study period. The choline intervention × time interaction term was not statistically significant (*P* = 0.27).

##### *d9*-PC

Significant main effects of the choline intervention (*P* = 0.034) were detected for maternal plasma *d9*-PC with lower plasma *d9*-PC enrichment observed in the intervention (vs. control) group throughout the study period. The choline intervention × time interaction term was not statistically significant (*P* = 0.107) (Figure 3C).

##### *d3*:*d9*-PC

Significant main effects of the intervention (*P* = 0.000013), time (*P* < 0.0001), and their interaction (*P* = 0.04; choline intervention × time) were detected for maternal plasma *d3*:*d9*-PC (Figure 3D). A higher ratio of *d3:d9-*PC enrichment was observed in the intervention (vs. control) group at GW 20–24 [0.79 (95% CI: 0.69, 0.89) vs. 0.53 (95% CI: 0.43, 0.63); *P* = 0.0005], GW 28–32 [0.92 (95% CI: 0.82, 1.01) vs. 0.54 (95% CI: 0.45, 0.65); *P* < 0.0001], and delivery [1.05% (95% CI: 0.95, 1.15) vs. 0.68% (95% CI: 0.58, 0.78); *P* < 0.0001].

#### Maternal lipid parameters

Because of choline's role in hepatic lipid export (including DHA), we assessed the effect of the choline intervention on lipid parameters in serum (Supplemental Table 2). Although no differences were detected between groups for HDL cholesterol (*P* = 0.4) and triglycerides (*P* = 0.8), LDL cholesterol (a derivative of VLDL cholesterol) tended to be elevated in the choline intervention (vs. control) group (main effect of intervention; *P* = 0.07).

## Discussion

The findings of this randomized controlled trial reveal that prenatal choline supplementation (administered across the second and third trimesters of pregnancy) significantly improves biomarkers of maternal DHA status among free-living participants consuming supplemental DHA. Higher concentrations of RBC PC-DHA tended to occur at GW 28–32 and higher concentrations of plasma PC-DHA and plasma total DHA, as well as RBC total DHA, were observed by GW 28–32 and were sustained through delivery among pregnant participants consuming habitual diets supplemented with 550 mg choline/d + 200 mg DHA/d, compared with those supplemented with 25 mg choline/d + 200 mg DHA/d. The higher circulating plasma PC-DHA, along with greater enrichments of both plasma *d3*-PC and *d3*-PC:*d9*-PC in the choline intervention arm (compared with control), strongly support the notion that prenatal choline chloride supplementation, a water-soluble choline salt, facilitates greater PEMT activity (and generation of PC molecules enriched in DHA) by bolstering methyl group supply.

Our finding of higher maternal DHA and PC-DHA in response to choline supplementation is consistent with previous work from our research group conducted in women of reproductive age whereby higher plasma and RBC PC-DHA were reported among those consuming controlled diets containing 930 mg choline/d + 200 mg DHA as compared with 480 mg choline/d + 200 mg DHA/d (7). However, our current findings deviate from the pregnant cohort of this study (7), whereby administration of 930 mg choline/d + 200 mg DHA/d across only the third trimester of pregnancy did not affect PC-DHA in either RBCs or plasma relative to 480 mg choline/d + 200 mg DHA/d (7). Two factors distinguish the current and past study—namely, the timing of choline supplementation, with the current intervention earlier in pregnancy, as well as the control group of the current intervention consuming habitual choline intakes, which were substantially lower than 480 mg. While both factors may have influenced PEMT activity, the current investigation observed strong choline supplementation effects on DHA outcomes at GW 28–32, shortly after the initiation of supplementation (∼GW 27) in West et al. (7), highlighting the importance of considering key metabolic windows in pregnancy.

Although no statistically significant effects of prenatal choline on newborn biomarkers of DHA status were detected, the overall pattern in magnitude was comparable to that observed in maternal blood. Indeed, cord blood RBC-DHA concentrations in the intervention arm were 0.41% higher than in the control arm, a finding that approached statistical significance (*P* = 0.12). The lack of significance may be due, in part, to a slightly smaller sample size of the newborn cohort, but also due to differences in metabolic priorities between the mother (prioritizes nutrient delivery) and her developing fetus (prioritizes cellular uptake and metabolic use). Indeed, while we and others have observed a decline in maternal plasma concentrations of PC-DHA and total DHA across the last trimester (7, 30, 31), indicative of significant DHA transport to the fetal compartment, the minimal change in cord RBC-DHA concentrations likely reflects the accretion of DHA in developing tissues, such as the brain [as previously shown in mice (32)], limiting our ability to observe an impact of prenatal choline supplementation on DHA status indicators in newborn RBCs.

Our investigation contains notable strengths and limitations. Strengths included *1*) the randomized, double-blind nature of this study spanning a large majority of gestation in women consuming typical choline intakes; *2*) measuring both physiological mediators (i.e., PC-DHA) and validated status indicators (i.e., RBC total DHA); and *3*) including stable isotope tracing, which represents a translational approach to the mechanistic physiology of one-carbon and lipid metabolism in pregnancy. Indeed, the overall pattern in DHA outcomes and isotopic labeling scheme present a concordant model by which habitual choline intakes limit, and choline supplementation improves, hepatic metabolism and DHA status across pregnancy. Further, it is a likely strength that these effects were observed despite high (>5%) baseline RBC total DHA status in this cohort, a characteristic typically expected to diminish the effect size of factors influencing additional DHA incorporation into membranes. It is a notable limitation, however, that we observed a nonsignificant (*P* = 0.105) effect of choline supplementation on our primary outcome of RBC PC-DHA. This may be due to issues of sample size and power, as RBC PC-DHA concentrations were significantly more variable than RBC total DHA (%CV: 18–27% vs. 9–15% across visits) and 2 significant outliers were present at delivery, a nonfasted time point, that impacted model estimates. However, RBC PC-DHA may be an undesirable outcome for physiological reasons as well. For example, it is notable that, in this cohort, RBC total DHA concentrations were maintained from visit 3 to delivery, whereas RBC PC-DHA exhibited a precipitous decline. The physiological basis for the significant redistribution of DHA from PC to other phospholipid pools in RBCs by the time of delivery, maintaining total DHA concentrations but decreasing the PC-DHA fraction, remains unclear. Our primary outcome choice was driven by available data at the time; however, our results make evident that plasma concentrations of PC-DHA, as well as RBC total DHA, are more sensitive to the impacts of choline supplementation, and should inform future studies.

There are notable inferences we cannot make from our study design. While we compared 2 doses of supplemental choline, informed by previously observed cognitive benefits (14), we did not have a third intermediate group to assess the dose–response relation between choline supplementation and DHA outcomes. Additionally, while our isotopic enrichment data support a clear role for the PEMT pathway metabolites in improving DHA status, there are notable nuances to the interpretation of the labeled choline metabolome in the context of an intervention that alters endogenous pool sizes across pregnancy, a physiological state that violates steady state assumptions, which we have detailed elsewhere (27).

Our results collectively indicate a significant nutrient–nutrient interaction between supplemental choline and supplemental DHA during pregnancy that warrants reflection on the large body of randomized trial evidence that has attempted to test the hypothesis that prenatal DHA supplementation improves fetal outcomes (33). Indeed, despite similar DHA intakes, we observed a nearly 75% relative increase in maternal DHA status in the intervention arm compared with the control. These data strongly suggest that prenatal choline supplementation influences the efficiency of hepatic DHA handling, mobilization, and ultimately, extrahepatic utilization, and support the view that existing clinical trials of prenatal DHA supplementation likely achieved non-maximal status, resulting from a limited methyl donor supply. As the field of nutrition pushes towards more “precision” approaches in hopes of identifying factors that influence the physiological response to nutrition exposures, accounting for known determinants of DHA response to supplementation during pregnancy, such as choline intake, will be key for understanding the causal, dose–response relations between DHA and maternal–infant outcomes.

## Supplementary Material

nqac147_Supplemental_File

## Data Availability

Data described in the manuscript, code book, and analytic code will be made available upon request pending request review and approval. Data sharing is dependent upon the nature of request and its compliance with approved data uses by the Institutional Review Board and associated informed consent.

## Notes

Supported by Balchem Corporation; the Cornell Institute of Biotechnology's Center for Advanced Technology (CAT) grant through New York State Division of Science, Technology, and Innovation (NYSTAR); and the National Institute of Food and Agriculture US Department of Agriculture (NIFA/USDA), HATCH, under accession number 1013729. The salary for KCK was provided from an NIH Training Grant (T32HD087137; T32ES027801). The funding sources had no role in the study design, interpretation of the data, or publication of results.

Author disclosures: The authors report no conflicts of interest. KCK is a member of the editorial board of the *American Journal of Clinical Nutrition* and played no role in the evaluation of this manuscript.

Supplemental Tables 1–5 are available from the “Supplementary data” link in the online posting of the article and from the same link in the online table of contents at https://academic.oup.com/ajcn/.

MQM is deceased.

Abbreviations used: ALT, alanine aminotransferase; ASA24, National Cancer Institute's Automated Self-Administered 24-hour Dietary Recall; AST, aspartate aminotransferase; *d0*-choline, unlabeled choline; *d9*-choline, methyl-*d9*-choline; DHQ-III, NIH Diet History Questionnaire; FFQ, food-frequency questionnaire; GLC, gas–liquid chromatography; GW, gestational week; HMRU, Human Metabolic Research Unit; ITT, intention to treat; LC-MS/MS, liquid chromatography–tandem mass spectrometry; LPC, lysophosphatidylcholine; MP, mobile phase; PC, phosphatidylcholine; PEMT, phosphatidylethanolamine *N*-methyltransferase; SAM, *S*-adenosylmethionine; WBC, white blood cell.
