# Supplementary material for: Prenatal choline supplementation improves biomarkers of maternal docosahexaenoic acid (DHA) status among pregnant participants consuming supplemental DHA: a randomized controlled trial
Source: Am J Clin Nutr. 2022 May 16;116(3):820–32. doi: 10.1093/ajcn/nqac147 (PMC9437984; doi:10.1093/ajcn/nqac147)
Supplement: nqac147_Supplemental_File [file nqac147_supplemental_file.docx]

# **ONLINE SUPPLEMENTARY MATERIAL**

**Prenatal choline supplementation improves biomarkers of maternal docosahexaenoic acid status among pregnant participants consuming supplemental DHA: a randomized controlled trial. Klatt, KC. et al.**

**Supplementary Table 1. In-Trial Events**

**Supplementary Table 2: Maternal Clinical Parameters**

**Supplementary Table 3: Infant Characteristics**

**Supplementary Table 4. Self-Reported Dietary Intakes**

**Supplementary Table 5. Sensitivity Analyses**

**Supplementary Table 1**. In-Trial Events

| **Intervention (n=17)** | **Control (n=16)** |
| --- | --- |
| Broken ankle (n=1) | Bronchitis with ear/sinus infection (n=1) |
| Dehydration (n=1) | Elevated liver enzymes; cholestasis (final week of pregnancy) (n=1) |
| Gestational hypertension (n=1) | Gestational hypertension (n=1) |
| Gestational diabetes mellitus (n=2) | Gestational diabetes mellitus (n=1) |
| Maternal anemia (n=1) | Hypothyroidism (n=1) |
| Maternal depression (n=1) | Placenta previa (n=1) |
| Single umbilical artery (n=1) | Sinus infection (n=1) |
| Sinus infection (n=1) |  |
| Total: n=9 | Total: n= 7 |

**Supplementary Table 2.** Maternal Clinical Parameters^1^

|  | Intervention  (n=15) | Control  (n=15) |
| --- | --- | --- |
| Hemoglobin, g/dL  Mean (95% CI) |  |  |
| *Visit 1* | 12.5 | 12.5 |
| *Visit 2* | 12.1 (11.6-12.6) | 12.1 (11.6-12.6) |
| *Visit* 3 | 11.8 (11.3-12.3) | 12.4 (11.9-12.9) |
| RBC, 10^^3^/uL  Mean (95% CI) |  | |
| *Visit 1* | 4.21 | 4.21 |
| *Visit 2* | 4.03 (3.87-4.19) | 4.00 (3.84-4.16) |
| *Visit* 3 | 4.04 (3.88-4.2) | 4.24 (4.08-4.4) |
| WBC, 10^^3^/uL  Mean (95% CI) |  | |
| *Visit 1* | 8.54 | 8.54 |
| *Visit 2* | 9.3 (8.47-10.12) | 9.79 (8.96-10.61) |
| *Visit* 3 | 8.79 (7.97-9.62) | 10.11 (9.28-10.93) |
| AST, U/L  Mean (95% CI) |  | |
| *Visit 1* | 18.4 | 18.4 |
| *Visit 3* | 18.8 (15.2-22.4) | 19.7 (16.2-23.3) |
| *Delivery* | 25.9 (22.2-29.6) | 23.4 (19.8-26.9) |
| ALT, U/L  Mean (95% CI) |  | |
| *Visit 1* | 29.2 | 29.2 |
| *Visit 3* | 24.7 (20.8-28.6) | 24.4 (20.5-28.3) |
| *Delivery* | 26.1 (22.1-30.2) | 22.5 (18.6-26.4) |

| Albumin, g/dL  Mean (95% CI) |  | |
| --- | --- | --- |
| *Visit 1* | 3.52 | 3.52 |
| *Visit 3* | 2.94 (2.8-3.08) | 2.91 (2.77-3.05) |
| *Delivery* | 2.69 (2.55-2.84) | 2.69 (2.55-2.83) |
| Glucose, mg/dL  Mean (95% CI) |  | |
| *Visit 1* | 79.7 | 79.7 |
| *Visit 3* | 79.7 (69.5-89.9) | 79.3 (69.1-89.5) |
| *Delivery* | 90.1 (79.5-100.7) | 95.3 (85.1-105.5) |
| Cholesterol, mg/dL  Mean (95% CI) |  | |
| *Visit 1* | 213 | 213 |
| *Visit 3* | 287 (254-320) | 267 (234-300) |
| *Delivery* | 278 (244-311) | 238 (205-271) |
| LDL, mg/dL  Mean (95% CI) |  | |
| *Visit 1* | 113 | 113 |
| *Visit 3* | 177 (152-203) | 159 (133-184) |
| *Delivery* | 170 (144-195) | 132 (107-158) |
| HDL, mg/dL  Mean (95% CI) |  | |
| *Visit 1* | 80.7 | 80.7 |
| *Visit 3* | 81.2 (70.7-91.7) | 75.8 (65.3-86.2) |
| *Delivery* | 71.4 (60.6-82.3) | 69.0 (58.5-79.4) |

| Triglycerides, mg/dL  Mean (95% CI) |  | |
| --- | --- | --- |
| *Visit 1* | 95.8 | 95.8 |
| *Visit 3* | 196 (167-226) | 192 (163-221) |
| *Delivery* | 201 (171-232) | 193 (164-222) |

^1^Visit 1 values reported represent the estimated marginal means for the baseline value adjusted for in the models.

.

**Supplementary Table 3.** Infant Characteristics

| Count/Arithmetic Mean | Intervention  (n=15) | Control  (n=15) | P-Value |
| --- | --- | --- | --- |
| Infant Sex, male  Count (%) | 6 (40%) | 4 (27%) | 0.4 |
| Birthweight, kg  Mean (SD) | 3.3 (0.43) | 3.43 (0.35) | 0.4 |
| Length, in  Mean (SD) | 48.92 (3.07) | 49.53 2.51) | 0.8 |
| Head circumference, in  Mean (SD) | 34.47 (1.19) | 34.24 (1.04) | 0.7 |
| Placental weight, g  Mean (SD) | 488 (75) | 515 (84) | 0.4 |
| Infant race, white  Count (%) | 11 (73%) | 15 (100%) | 0.10 |
| APGAR score  Mean (SD) | 9 (0) | 8 (2) | 0.12 |

**Supplementary Table 4.** Self-Reported Dietary Intakes

|  |  | **Baseline ASA24 Dietary Recall (n=28)** | | | | | | | | | | | |
| --- | --- | --- | --- | --- | --- | --- | --- | --- | --- | --- | --- | --- | --- |
|  |  | **Intervention  (n=14)** | | | | | **Control  (n=14)** | | | | |  |  |
| **Nutrient** |  | **Mean** | **SD** | **Median** | **Q1** | **Q3** | **Mean** | **SD** | **Median** | **Q1_** | **Q3** | **ProbT^1^** | **ProbChi^2^** |
| **ARA (mg)** |  | 150.8 | 129.7 | 139.0 | 44.9 | 186.0 | 148.0 | 95.3 | 153.6 | 82.2 | 238.6 | 0.95 | 0.82 |
| **Carbohydrates (g)** |  | 237.4 | 57.7 | 224.5 | 194.7 | 291.5 | 240.3 | 74.2 | 243.1 | 199.1 | 280.1 | 0.91 | 0.93 |
| **Cholesterol (mg)** |  | 325.2 | 224.4 | 277.4 | 171.0 | 377.3 | 329.2 | 196.1 | 250.4 | 181.2 | 507.1 | 0.96 | 1.00 |
| **Choline (mg)** |  | 346.5 | 184.5 | 320.3 | 204.6 | 384.0 | 383.0 | 117.2 | 363.7 | 325.8 | 505.6 | 0.54 | 0.27 |
| **DHA (mg)** |  | 56.7 | 99.9 | 14.4 | 1.2 | 85.2 | 43.9 | 40.9 | 36.4 | 10.0 | 70.5 | 0.66 | 0.76 |
| **EPA (mg)** |  | 17.3 | 43.2 | 2.4 | 0.3 | 3.5 | 11.5 | 12.4 | 7.8 | 2.8 | 16.7 | 0.64 | 0.07 |
| **Folic acid (mcg)** |  | 188.9 | 124.0 | 157.5 | 113.8 | 209.7 | 232.7 | 174.4 | 171.6 | 120.0 | 308.6 | 0.45 | 0.61 |
| **Folate*DFE (mcg)** |  | 587.6 | 229.1 | 626.4 | 434.3 | 656.6 | 704.3 | 328.8 | 576.9 | 443.3 | 910.9 | 0.29 | 0.49 |
| **Food folate (mcg)** |  | 266.9 | 89.4 | 281.3 | 199.0 | 311.6 | 309.0 | 85.0 | 303.4 | 264.6 | 386.2 | 0.21 | 0.27 |
| **Folate (mcg)** |  | 455.7 | 152.5 | 487.8 | 339.5 | 548.4 | 541.7 | 212.7 | 483.1 | 381.1 | 694.7 | 0.23 | 0.46 |
| **Iron (mg)** |  | 14.5 | 3.2 | 14.4 | 11.7 | 17.4 | 17.8 | 6.3 | 15.7 | 12.9 | 23.5 | 0.10 | 0.29 |
| **Calories (kcal)** |  | 2017.5 | 482.9 | 2033.1 | 1575.5 | 2322.4 | 2178.5 | 454.7 | 2180.7 | 1751.0 | 2489.4 | 0.37 | 0.41 |
| **Protein (g)** |  | 81.5 | 31.7 | 72.0 | 58.5 | 95.0 | 95.6 | 17.8 | 92.1 | 82.2 | 108.1 | 0.16 | 0.05 |
| **Fat (g)** |  | 86.3 | 25.7 | 85.2 | 62.5 | 111.6 | 98.6 | 30.5 | 85.9 | 74.5 | 122.5 | 0.26 | 0.33 |
| **Vitamin B12 (mcg)** |  | 4.1 | 2.1 | 4.1 | 3.1 | 4.6 | 5.1 | 3.0 | 4.5 | 3.6 | 5.2 | 0.30 | 0.23 |
| **Vitamin B6 (mg)** |  | 1.8 | 0.7 | 1.8 | 1.3 | 2.1 | 2.4 | 0.9 | 2.2 | 1.9 | 2.9 | 0.05 | 0.04 |
|  | | | | | | | | | | | | | |

|  | **Throughout Study ASA24 Dietary Recalls (n=102)** | | | | | | | | | | | |
| --- | --- | --- | --- | --- | --- | --- | --- | --- | --- | --- | --- | --- |
|  | **Intervention  (n=14)** | | | | | **Control  (n=15)** | | | | |  |  |
| **Nutrient** | **Mean** | **SD** | **Median** | **Q1** | **Q3** | **Mean** | **SD** | **Median** | **Q1_** | **Q3** | **ProbT^1^** | **ProbChi^2^** |
| **ARA (mg)** | 119.3 | 62.8 | 96.1 | 62.5 | 143.4 | 182.5 | 93.3 | 171.3 | 69.4 | 227.5 | 0.04 | 0.10 |
| **Carbohydrates (g)** | 243.8 | 49.6 | 248.3 | 205.3 | 279.1 | 271.9 | 92.0 | 254.7 | 220.8 | 314.3 | 0.31 | 0.34 |
| **Cholesterol (mg)** | 280.1 | 116.5 | 247.8 | 151.9 | 333.3 | 365.1 | 168.6 | 298.2 | 232.4 | 386.1 | 0.12 | 0.22 |
| **Choline (mg)** | 337.2 | 65.7 | 353.1 | 253.4 | 372.9 | 401.8 | 119.5 | 376.9 | 321.1 | 420.6 | 0.08 | 0.15 |
| **DHA (mg)** | 84.0 | 105.0 | 17.3 | 5.3 | 43.3 | 74.0 | 121.1 | 33.7 | 9.6 | 53.9 | 0.81 | 0.41 |
| **EPA (mg)** | 36.9 | 56.7 | 5.2 | 2.6 | 6.8 | 31.9 | 66.1 | 6.0 | 4.9 | 9.2 | 0.83 | 0.19 |
| **Folic acid (mcg)** | 198.7 | 78.7 | 171.4 | 124.9 | 256.1 | 204.9 | 97.0 | 190.9 | 152.9 | 254.3 | 0.85 | 0.76 |
| **Folate*DFE (mcg)** | 608.3 | 190.0 | 580.5 | 474.1 | 623.8 | 709.0 | 223.5 | 685.9 | 598.5 | 842.6 | 0.20 | 0.14 |
| **Food folate (mcg)** | 270.6 | 91.7 | 237.6 | 176.2 | 336.8 | 360.7 | 115.4 | 329.7 | 234.5 | 487.7 | 0.03 | 0.09 |
| **Folate (mcg)** | 469.2 | 142.6 | 446.6 | 374.1 | 492.6 | 565.5 | 168.2 | 519.3 | 425.9 | 663.0 | 0.11 | 0.10 |
| **Iron (mg)** | 15.1 | 2.7 | 14.3 | 14.0 | 14.9 | 17.6 | 4.2 | 19.0 | 13.2 | 20.0 | 0.07 | 0.10 |
| **Calories (kcal)** | 1968.2 | 266.1 | 1954.0 | 1853.2 | 2124.0 | 2311.8 | 666.8 | 2297.2 | 1581.4 | 2874.4 | 0.08 | 0.12 |
| **Protein (g)** | 83.1 | 13.2 | 76.7 | 67.9 | 91.1 | 98.5 | 27.9 | 92.4 | 75.3 | 110.6 | 0.07 | 0.06 |
| **Fat (g)** | 78.2 | 15.0 | 73.7 | 65.6 | 86.0 | 97.9 | 35.6 | 98.9 | 63.6 | 134.3 | 0.06 | 0.16 |
| **Vitamin B12 (mcg)** | 5.3 | 2.3 | 4.7 | 4.1 | 5.8 | 5.5 | 2.4 | 4.5 | 3.1 | 7.5 | 0.86 | 0.96 |
| **Vitamin B6 (mg)** | 2.3 | 0.9 | 2.0 | 1.9 | 2.6 | 2.6 | 0.7 | 2.5 | 1.9 | 3.4 | 0.27 | 0.24 |

^1^Satterthwait estimate from t-test of means between treatment groups

^2^p-value from Hodges-Lehman-Sen estimation (nonparametric comparison of medians)

# **Supplementary Table 5**: Sensitivity Analyses of the effect of prenatal choline supplementation on maternal RBC total DHA and maternal plasma total DHA among pregnant participants consuming 200 mg supplemental DHA/d.

|  | **Unadjusted ^1^** | | | | | |
| --- | --- | --- | --- | --- | --- | --- |
|  | Intervention  *n = 15* | Control  *n = 15* | P-value | Intervention  *n = 15* | Control  *n = 15* | P-value |
| ***Study Week*** | Mean  (95% CI) | Mean  (95% CI) |  | Mean  (95% CI) | Mean  (95% CI) |  |
| Visit 1  *GW 12-16* | 6.1  (5.7-6.5) | 5.9  (5.5-6.3) | 0.46 | 100  (85-115) | 78  (63-93) | 0.26 |
| Visit 2  *GW 20-24* | 7.2  (6.7-7.6) | 6.9  (6.5-7.4) | 0.36 | 134  (119-149) | 112  (97-127) | 0.18 |
| Visit 3  *GW 28-32* | 7.9  (7.5-8.3) | 7.2  (6.8-7.7) | 0.016 | 143  (128-158) | 121  (106-136) | 0.006 |
| Delivery | 8.0  (7.6-8.5) | 7.1  (6.7-7.6) | 0.002 | 122  (107-137) | 100  (85-115) | 0.0008 |
|  | **Fully Adjusted ^2^** | | | | | |
|  | Intervention  *n = 15* | Control  *n = 15* | P-value | Intervention  *n = 15* | Control  *n = 15* | P-value |
| ***Study Week*** | Mean  95% CI | Mean  95% CI |  | Mean  95% CI | Mean  95% CI |  |
| Visit 1  *GW 12-16* | 6.0 | 6.0 |  | 89 | 89 |  |
| Visit 2  *GW 20-24* | 7.1  (6.7-7.4) | 7.0  (6.7-7.4) | 0.9 | 125  (109-140) | 122  (107-137) | 0.79 |
| Visit 3  *GW 28-32* | 7.8  (7.4-8.2) | 7.4  (7.0-7.7) | 0.08 | 141  (126-156) | 123  (108-138) | 0.08 |
| Delivery | 7.9  (7.5-8.3) | 7.2  (6.9-7.6) | 0.01 | 123  (108-139) | 99  (84-114) | 0.02 |

^1^Model includes significant time x intervention interaction terms.

^2^Model includes baseline model parameters as well as age, pre-pregnancy BMI, and intervention duration.
